# Learning-induced remodelling of inhibitory synapses in the motor cortex

**DOI:** 10.1098/rsob.240109

**Published:** 2024-11-13

**Authors:** Nishita Bhembre, Annalisa Paolino, Sooraj S. Das, Sumasri Guntupalli, Laura R. Fenlon, Victor Anggono

**Affiliations:** ^1^ Clem Jones Centre for Ageing Dementia Research, Queensland Brain Institute, University of Queensland, Brisbane, Queensland 4072, Australia; ^2^ School of Biomedical Sciences, Faculty of Medicine, University of Queensland, Brisbane, Queensland 4072, Australia

**Keywords:** learning, plasticity, inhibition, postsynaptic density, gephyrin

## Abstract

Robust structural and functional plasticity occurs at excitatory synapses in the motor cortex in response to learning. It is well established that local spinogenesis and the subsequent maintenance of newly formed spines are crucial for motor learning. However, despite local synaptic inhibition being essential for shaping excitatory synaptic input, less is known about the structural rearrangement of inhibitory synapses following learning. In this study, we co-expressed the structural marker tdTomato and a mEmerald-tagged intrabody against gephyrin to visualize inhibitory synapses in layer 2/3 cortical neurons of wild-type CD1 mice. We found that a 1-day accelerated rotarod paradigm induced robust motor learning in male and female adult CD1 mice. Histological analyses revealed a significant increase in the surface area of gephyrin puncta in neurons within the motor cortex but not in the somatosensory cortex upon motor learning. Furthermore, this learning-induced reorganization of inhibitory synapses only occurred in dendritic shafts and not in the spines. These data suggest that learning induces experience-dependent remodelling of existing inhibitory synapses to fine-tune intrinsic plasticity and input-specific modulation of excitatory connections in the motor cortex.

## 1. Introduction

The precise arrangement of neurotransmitter receptors at the postsynaptic membrane is crucial for efficient synaptic transmission and plasticity. Modular scaffolding molecules such as postsynaptic density protein 95 (PSD-95) and gephyrin are critical in anchoring and clustering neurotransmitter receptors, concentrating these receptors at excitatory and inhibitory synapses, respectively [1–3]. Activity-dependent remodelling of postsynaptic scaffolding molecules contributes to the plasticity of functional synapses during development and in response to changes in patterns of neural activity or sensory experiences [4,5]. This is particularly evident in excitatory synapses, where dendritic spines undergo extensive structural remodelling during synaptic plasticity and learning [6–13]. It is well established that the spine volume positively correlates with the area of PSD and α-amino-3-hydroxy-5-methyl-4-isoxazole propionic acid (AMPA) receptor content and, hence, synaptic strength [8,10,14,15]. Although stimulated spines undergo rapid growth, the addition of PSD-95 molecules occurs at a slower time scale [16–19]. The gradual increase of PSD-95 in size and complexity promotes spine stability and precise alignment with presynaptic structures [19–23].

Accumulating evidence has demonstrated that the plasticity of inhibitory synapses plays essential roles in fine-tuning synaptic efficacy, refining neural circuitry and maintaining network synchronization and stability [24,25]. Major inhibitory neurotransmitter receptors, including γ-aminobutyric acid type-A (GABA_A_) and glycine receptors, are clustered at the synapse and anchored to the cytoskeleton by the postsynaptic scaffolding molecule, gephyrin [3,26]. Similar to PSD-95 and AMPA receptors, gephyrin and GABA_A_ receptors are organized into subsynaptic nanodomains in neurons [27–29]. Activity-dependent redistribution of gephyrin molecules into and out of inhibitory PSDs is a major regulatory process that determines the dynamic changes in the strength of inhibitory neurotransmission during bidirectional synaptic plasticity [30–36]. Importantly, long-term *in vivo* imaging of inhibitory synapses in the layer 2/3 neurons of the mouse visual cortex has also revealed the dynamic nature of gephyrin clusters that continuously assemble and dissemble on the dendritic shaft and dually innervated dendritic spines [37–39]. However, little is known about the structural reorganization of inhibitory synapses in the context of learning.

Here, we expressed a genetically encoded fibronectin intrabody generated by mRNA display (FingR) against gephyrin [40], fused with the fluorescent protein mEmerald [41], to label inhibitory synapses along the dendritic regions of layer 2/3 cortical neurons in CD1 mice. We found that a 1-day rotarod motor learning task induced a robust increase in the size of gephyrin puncta in the dendritic shaft but not in the dendritic spine of neurons in the motor cortex. The overall gephyrin puncta density was not altered. Importantly, these effects were only observed in the motor cortex and not in the somatosensory cortex of the trained mice. Taken together, our results demonstrate that the PSD of inhibitory synapses undergoes robust structural remodelling following motor skill acquisition, which has important implications in neuronal plasticity, and the formation and refinement of the neural circuitry underlying learned movements.

## 2. Results

### 2.1. Visualization of endogenous gephyrin puncta in cortical neurons of CD1 mice

**Figure 1.**
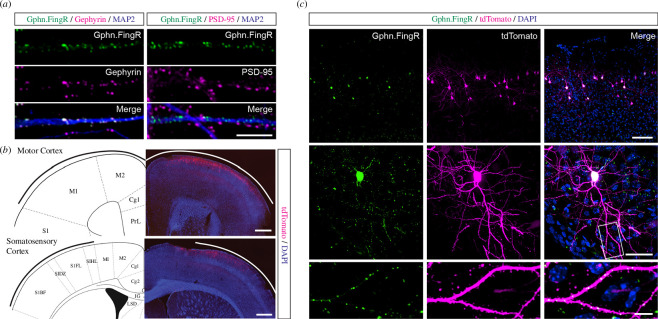
Visualization of inhibitory synapses with Gphn.FingR–mEmerald *in vitro* and *in vivo*. (*a*) Representative images of primary hippocampal neurons expressing the Gphn.FingR–mEmerald intrabodies (green) that were immunostained with specific antibodies against MAP2 (blue) and gephyrin (magenta, left panels) or PSD-95 (magenta, right panels). Scale bar, 10 μm. (*b*) Representative coronal sections of *in utero* electroporated patches labelling layer 2/3 neurons of the motor (upper panels) and somatosensory cortices (lower panels). DAPI staining is shown in blue. Scale bar, 100 μm. Reference mouse brain atlases were modified from Franklin & Paxinos [44]. (*c*) Representative images of neurons expressing Gphn.FingR–mEmerald (green) and the structural marker tdTomato (magenta) at 40× (top panels) and 60× (middle panels) magnifications from the M1 motor cortex. The bottom panels show zoomed-in images of a dendritic segment of the neuron in the middle panel (white box). DAPI staining is shown in blue. Scale bar, 100 μm (top panels), 50 μm (middle panels) and 10 μm (bottom panels).

Fluorescent protein tagging is the most conventional method for investigating protein localization and dynamics in cells. However, overexpression of scaffolding proteins in the PSD often results in the alteration of synaptic function. For example, overexpression of PSD-95 causes enhanced maturation of excitatory synapses by increasing the size and numbers of dendritic spines and synaptic AMPA receptors [42]. Likewise, overexpression of gephyrin has also been reported to affect the density and size of gephyrin puncta and glutamatergic synapses [43]. To avoid these undesirable side effects, we visualized inhibitory synapses by expressing a recombinant FingR intrabody that recognizes endogenous gephyrin (Gphn.FingR) and fused it with a fluorescent protein [40], as it is well established that overexpression of Gphn.FingR does not affect the size of the gephyrin puncta and the electrophysiological properties of neurons [40]. To enhance the brightness of the existing green fluorescent protein (GFP)-tagged Gphn.FingR, we generated a construct that encodes Gphn.FingR–mEmerald fusion protein on a vector that contains the CCR5 zinc finger and KRAB(A) repressor transcriptional control system [40]. When transfected into primary hippocampal neurons in culture, we observed perfect colocalization of Gphn.FingR–mEmerald–CCR5TC with endogenous gephyrin but not with PSD-95 puncta, which confirmed the specificity of this construct (figure 1*a*).

To visualize gephyrin-positive inhibitory synapses *in vivo*, we co-expressed two plasmids that encode Gphn.FingR–mEmerald–CCR5TC and the structural marker tdTomato via *in utero* electroporation in CD1 mice at embryonic day 15 (E15). Histological analyses of the adult electroporated mice validated the expression of these constructs in layer 2/3 neurons in the motor and somatosensory cortices (figure 1*b*). Moreover, the visualization of these histological sections on a spinning disc confocal microscope with higher magnification revealed that most of the Gphn.FingR–mEmerald–CCR5TC puncta reside along the dendritic shaft (figure 1*c*). Unbound intrabodies, which were in excess, localized in the nucleus, where they turn off the transcription of new transcripts encoding Gphn.FingR (figure 1*c*). These data demonstrate the utility of the Gphn.FingR–mEmerald–CCR5TC construct to mark inhibitory synapses both *in vitro* and *in vivo*, which is consistent with results reported in a recent study that used Gphn.FingR–mScarlett [45].

### 2.2. Motor learning induces structural remodelling of inhibitory synapses

**Figure 2.**
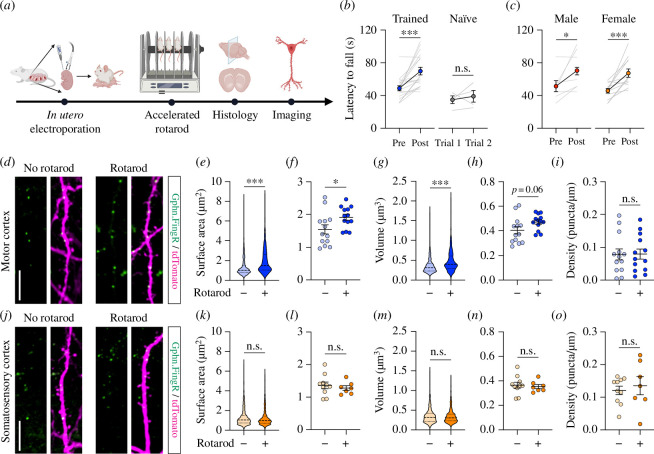
Accelerated rotarod learning increases the surface area of gephyrin puncta in the motor cortex. (*a*) A schematic of the experimental design. The image was created with BioRender.com. (*b*) Adult CD1 mice that underwent 1 day of accelerated rotarod training showed a significant increase in their latency to fall in trials conducted 24 h post-learning, compared to naive mice (training group, *n* = 19 mice; naive group, *n* = 4 mice). Grey lines represent the performance of individual mice before and after training (ratio paired *t*-test, ***p < 0.001, n.s. = not significant). (*c*) Both male and female CD1 mice effectively learned the accelerated rotarod task (male, *n* = 7 mice; female, *n* = 12 mice; ratio paired *t*-test, *p < 0.05, ***p < 0.001). (*d*) Confocal images of dendritic segments of neurons co-expressing Gphn.FingR–mEmerald (green) and tdTomato (magenta) in the motor cortex of naive (left panels) or trained (right panels) mice. Motor learning significantly increased the surface area of individual gephyrin puncta (*e*) and their average size across individual neurons (*f*). Quantification of the volume of individual gephyrin puncta (*g*) and the average across individual neurons (*h*). (*i*) Motor learning did not affect gephyrin puncta density. *n* = 1352 puncta and 13 neurons from 3 mice (no rotarod group) and 1039 puncta and 13 neurons from 4 mice (rotarod group). (*j*–*o*) The accelerated rotarod learning task did not alter the surface area (*k,l*), volume (*m,n*) or density (*o*) of gephyrin puncta in neurons within the somatosensory cortex. *n* = 1191 puncta and 10 neurons from three mice (no rotarod group) and 794 puncta and 7 neurons from four mice (rotarod group). All data represent mean ± s.e.m., except for the violin plots, which show the median and quartile values. Data were subjected to Mann–Whitney (*e,g,k,m*) or unpaired *t*-test (*f,h,i,l,n,o*), *p < 0.05, ***p < 0.001, n.s. = not significant). Scale bars, (*d*,*j*) 10 μm.

To study the effect of acute learning on the structure of inhibitory synapses, we quantified the endogenous gephyrin puncta in layer 2/3 cortical neurons of *in utero* electroporated CD1 mice co-expressing Gphn.FingR–mEmerald–CCR5TC and tdTomato (figure 2*a*). We chose motor learning as an experimental model due to the presence of excitatory synapse remodelling and dendritic spine dynamics in the motor cortex and their essential roles for improved motor performance after learning [11–13,46]. We adopted the accelerated rotarod task in which mice were trained to maintain their balance on a rotating rod with increasing acceleration over 20 sessions in a single day. At the end of the training sessions, the mice showed a significant increase in the latency to fall off the accelerated rotarod compared to untrained mice (figure 2*b*). This improved motor performance was observed in both genders (figure 2*c*), demonstrating that the single-day accelerated rotarod task is a robust motor skill learning paradigm in adult CD1 mice.

Next, we performed histological analyses on the brains of adult CD1 mice and quantified the number and size of Gphn.FingR–mEmerald puncta in the principal neurons within layer 2/3 of the motor cortex of the trained and untrained animals (figure 2*d*). We observed a significant increase in the surface area of individual gephyrin puncta (figure 2*e*) across multiple neurons (figure 2*f*) in the motor cortex of rotarod-trained mice, compared to naive animals. We also saw a significant increase in the volume of individual gephyrin puncta (figure 2*g*). However, it did not reach statistical significance when we compared the mean volume across neurons (*p* = 0.06, figure 2*h*). Despite the increase in the size of inhibitory synapses following motor learning, the number of gephyrin puncta did not appear to change (figure 2*i*). These effects were specific to neurons in the motor cortex as they were not observed in somatosensory cortical neurons (figure 2*j–o*).

**Figure 3.**
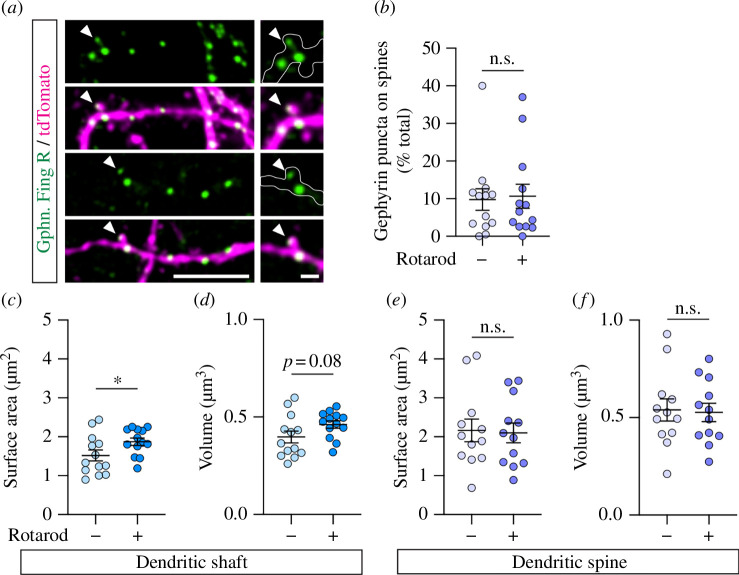
Motor learning does not significantly alter the surface area of gephyrin puncta in the dendritic spines. (*a*) Representative images of dendritic segments of neurons co-expressing Gphn.FingR–mEmerald (green) and tdTomato (magenta) in the motor cortex. Arrowheads indicate gephyrin puncta in dendritic spines. Scale bars, 10 μm and 2 μm (inset). (*b*) Accelerated rotarod learning did not affect the density of gephyrin puncta on dendritic spines. (*c*–*f*) Quantification of the surface area and volume of gephyrin puncta in dendritic shafts (*c*,*d*, *n* = 13 neurons per group) and spines (*e*,*f*, *n* = 12 neurons per group). All data represent mean ± s.e.m. (unpaired *t*-test, *p < 0.05, n.s. = not significant).

Although most inhibitory synapses are found on the soma and dendritic shafts, inhibitory nerve terminals also innervate the dendritic spines of neocortical and hippocampal neurons [37,45,47,48]. Indeed, we were able to observe Gphn.FingR–mEmerald labelling within dendritic spines (figure 3*a*), which accounted for approximately 10% of the total gephyrin puncta in cortical neurons (figure 3*b*). To determine the effects of learning on inhibitory synapse remodelling in the dually innervated spines, we performed analyses of Gphn.FingR–mEmerald puncta on the dendritic shaft and spine separately. As expected, we found a significant increase in the surface area (figure 3*c*) and a trend towards larger volume (figure 3*d*, *p* = 0.08) of gephyrin puncta on the dendritic shafts of neurons in the motor cortex in response to acute motor learning. Although previous studies have shown that inhibitory synapses on spines are slightly more dynamic (assembled and removed) than shaft inhibitory synapses or dendritic spines, and that this process is responsive to sensory experience [37,38,49], we did not see any significant changes in the surface area or volume of gephyrin puncta on dendritic spines (figure 3*e*,*f*). However, we cannot exclude the possibility that transient changes of gephyrin dynamics in these dually innervated spines occur outside our observation point (24 hours) before or after motor learning, as this might allow flexible, input-specific modulation of stable excitatory synapses.

## 3. Discussion

It is well established that the structural reorganization of synapses and active remodelling of the PSD contribute to changes in neuronal plasticity, functional connectivity and circuit refinement during learning [9,50,51]. In the context of motor learning, the formation of spatially clustered dendritic spines provides a means for the local reorganization of the excitatory circuit in layer 2/3 of the motor cortex [13,46,52]. Widespread modulation of inhibitory neurons in the cortex during motor learning has also been reported [52,53]. In this study, we asked whether learning could induce structural remodelling of inhibitory PSDs in the motor cortex following an accelerated rotarod paradigm. By labelling endogenous gephyrin puncta with the genetically encoded intrabody Gphn.FingR–mEmerald, we found that motor learning increased the surface area of gephyrin puncta without affecting their overall density in the dendritic shaft of layer 2/3 neurons in the motor cortex. The learning-related changes in the size of inhibitory PSDs were system-specific as they did not occur in layer 2/3 neurons of the somatosensory cortex.

Previous studies have shown that cortical disinhibition contributes to the expression of structural plasticity in excitatory neurons [52–55], which is associated with a significant reduction in GABA release in the human primary motor cortex [56,57]. In rodents, cortical disinhibition during the initial learning phase is mediated by reduced inhibition by somatostatin- and parvalbumin-positive interneurons onto the apical dendrites and perisomatic regions of layer 2/3 motor neurons, respectively [52,54,55]. Our results might initially seem counterintuitive because the increase in gephyrin puncta size has previously been shown to correlate well with the strength of GABA_A_ receptor-mediated currents [30,31,34]. However, a more recent study has shown that an acute augmentation of gephyrin surface area disperses the localization of synaptic GABA_A_ receptors and disrupts the precise nanocolumn alignment at the inhibitory synapses, thereby reducing inhibitory synaptic neurotransmission [58]. The next logical step in testing this hypothesis is to visualize the nanoscale organization of GABA_A_ receptors within these enlarged gephyrin puncta associated with motor learning.

Cortical inhibition during learning is highly dynamic. After the initial learning phase, the excitatory drive onto parvalbumin-expressing interneurons increases [55]. This post-learning hyperexcitation may function to limit excessive structural plasticity and preserve learned information [59–61]. It could also contribute to restoring the balance between excitation and inhibition of the neural network [62]. These two functions are complementary and are not mutually exclusive. It is conceivable that the increase in gephyrin surface area following a single day of rotarod learning may contribute to enhanced cortical inhibition to subserve those functions. Therefore, it is critical that future experiments are focused on measuring inhibitory neurotransmission in the layer 2/3 cortical neurons in the motor cortex 24 hours post-rotarod learning.

In summary, our results provide empirical evidence for learning-induced structural remodelling of inhibitory synapses in the mouse motor cortex following a day of accelerated motor learning. These structural changes after short-term motor learning might reflect the learning-induced modulation of neuronal inhibition as an adaptive state of a dynamic cortical network. Our method should be applicable for future experiments to monitor the structural rearrangement of inhibitory PSDs during the initial learning phase and for tracking their dynamics over multiple days when combined with *in vivo* two-photon imaging in awake mice. As Gphn.FingR–mEmerald is a genetically encoded intrabody, it can be expressed in a distinct population of neurons with cell-specific promoters to gain deeper insights into the spatial and temporal regulation of structural plasticity of inhibitory PSDs in the brain during learning and memory processes.

## 4. Material and methods

### 4.1. Animals

Wild-type CD1 mice were purchased from the University of Queensland Biological Resources. Timed-mating was set up by placing CD1 male and female mice in the same cage overnight. When a vaginal plug was observed, it was considered the first embryonic day (E0). All animals were housed in a 20°C room on a 12 hour day-and-night cycle. Food and water were supplied ad libitum. All research procedures involving animal use were conducted according to the Australian Code of Practice for the Care and Use of Animals for Scientific Purposes and were approved by the University of Queensland Animal Ethics Committee (2021/AE000100).

### 4.2. DNA constructs

The pCAG-Gphn.FingR–mEmerald–CCR5TC construct was generated from pCAG–PSD-95.FingR–eGFP–CCR5TC (Addgene 46295) and pCAG–Gphn.FingR–mKate2–IL2RGTC (Addgene 46295), both of which were gifts from Don Arnold [40]. We first replaced the eGFP in pCAG–PSD-95.FingR–eGFP–CCR5TC to generate pCAG–PSD-95.FingR–mEmerald–CCR5TC. We then removed the PSD-95.FingR and subsequently subcloned Gphn.FingR into the parent vector to create pCAG–Gphn.FingR–mEmerald–CCR5TC. The pCAG–tdTomato construct was a gift from Patricio Opazo.

### 4.3. Antibodies

The following antibodies were obtained from commercial sources: mouse anti-gephyrin (Synaptic Systems Cat. no. 147 011, RRID:AB_887717, used at 1 : 50 dilution), chicken anti-MAP2 (Abcam Cat. no. ab92434, RRID:AB_2138147, used at 1 : 2000 dilution), and mouse anti-PSD-95 (BioLegend Cat. no. 810401, RRID:AB_2564750, used at 1 : 500 dilution). Alexa-conjugated secondary antibodies were purchased from Invitrogen and used at 1 : 500 dilution.

### 4.4. *In utero* electroporation

In order to label layer 2/3 neocortical neurons, time-mated CD1 pregnant dams were electroporated at embryonic stage (E) 15 for all experiments [63]. Briefly, the pregnant dams were anaesthetized with an intraperitoneal injection of ketamine (120 mg kg^−1^) and xylazine (10 mg kg^−1^). The embryos were then exposed from the abdominal cavity by laparotomy under sterile conditions. Each embryo was positioned so that the lateral telencephalic ventricles were visible through the uterine wall. One microlitre of plasmid solution (containing 0.5 μg of pCAG–Gphn.FingR–mEmerald–CCR5TC, 0.5 μg pCAG–tdTomato and 0.0025% Fast Green dye) was injected into the lateral ventricles with glass-pulled micropipettes and a Picospritzer. Five 100 ms square pulses of 30–35 V were delivered at 1 Hz with 1 to 3 mm paddles (ECM 803, BTX). Once this procedure was completed, the uterine horns were replaced inside the abdominal cavity, and the incision was sutured twice. Animals were then subcutaneously injected with 1 ml of sterile saline solution and recovered in a humidified chamber at approximately 28°C. For pain relief, an edible buprenorphine gel pack was prepared by injecting MediGel with 0.2 ml of buprenorphine solution (0.026 mg ml^−1^) and positioned next to the recovering dam in the cage. Dams were then monitored daily until they gave birth to live pups. The fluorescent patches in the cortex of pups at postnatal day 2 were checked using fluorescence goggles. Only pups with fluorescent patches were kept until they reached the desired experimental age.

### 4.5. Accelerated rotarod task

Adult CD1 mice (2–6 months old) were trained on an accelerated rotarod system (Ugo Basile^®^ SRL) to induce motor learning according to a protocol adapted from [11] and performed during daytime (light phase). Before training began, the mice were habituated to their cage and behaviour room for 30 min and then on a stationary rotarod apparatus for 2 min. During the training sessions, mice were first exposed to the fixed-speed protocol (8 r.p.m.), allowing them to acclimatize to the rotating movement as they rarely fall off the rod at this relatively low speed. Following this, the speed of the rotarod was increased stepwise from 4 to 60 r.p.m. over 2 min. During the training phase, the mice were gently tapped on the hind limbs with a plastic Pasteur pipette to teach them to face forward on the rotating rod, helping them to stay on the rotarod at higher speeds. After falling, the mice were immediately placed back on the rotarod. This 1-day protocol involved 20 training sessions, split into two training blocks with a 5 min interval. To assess motor learning, each mouse underwent three trials of the acceleration protocol (5 min inter-trial intervals), and the latency to fall was recorded.

### 4.6. Histology

Following deep anaesthesia, mice were transcardially perfused with 0.9% saline followed by a 4% paraformaldehyde (PFA) solution prepared in 0.1 M phosphate-buffered saline (PBS). The brains were post-fixed in 4% PFA solution for 48 hours and stored in 0.1 M PBS containing 1% sodium azide at 4°C. They were then embedded in 3.5% agarose and sectioned into 50 µm coronal slices using a vibratome (Leica VT 1000S). Coronal brain sections were collected and washed three times in 0.1 M PBS and stored in 0.1 M PBS containing 1% sodium azide at 4°C. They were then mounted on positively charged slides (UberFrost^®^, InstrumeC), dried and post-fixed with 4% PFA for 10 min. After washing with PBS, the mounted sections were stained with 0.1% 4′,6-diamidino-2′-phenylindole (DAPI) solution for 10 min, washed and coverslipped (Menzel-Gläser) with anti-fade mounting medium (Dako).

### 4.7. Microscopy and image analyses

Wide-field fluorescence imaging was performed using a Zeiss upright Axio-Imager Z1 fluorescence microscope on a 10× Plan-Apochromat objective. High-resolution fluorescence images were acquired with a Diskovery spinning disc confocal microscope at various magnifications using 40× water immersion and 60× oil immersion objectives. Individual dendrites from the 60× images were carefully traced for analysis using Neurolucida 360 software (MBF Bioscience, RRID:SCR_016788). The entire dendritic trees of individual neurons were reconstructed in three dimensions from the series of 50 µm coronal sections. Individual dendritic spines were semi-automatically detected and classified across all image traces using identical detection parameters (table 1). Similarly, individual gephyrin puncta were detected automatically in all traces using consistent parameters.

**Table 1.** Neurolucida 360 detection parameters for soma, dendritic tree and puncta detection.

parameters	soma	dendritic tree	puncta
detector sensitivity	50–70 a.u.	NA	100–300%
tracing/ detection mode	automatic	user-guided	automatic
size constraint/ minimum size	5 µm	NA	2 voxels
interactive search region	20 µm	NA	NA
detection diameter	NA	NA	1 µm
other	NA	— typical process width: 0.5 µm — method: directional kernels	— detect based on proximity to trees (0.5 µm) — filter image noise

### 4.8. Primary neuronal cultures and transfection

Sprague–Dawley rat embryos (E18) were used to prepare primary hippocampal neurons as described previously [64]. Briefly, hippocampi were isolated and dissociated with 30 U of papain suspension (Worthington) for 20 min in a 37°C water bath. A single-cell suspension was obtained by triturating tissues with fire-polished glass Pasteur pipettes and then plated at a density of 8 × 10^4^ cells on a poly-l-lysine-coated coverslip in Neurobasal growth medium supplemented with 2 mM Glutamax, 1% penicillin/streptomycin and 2% B-27. Neurons were then maintained in a humidified 5% CO_2_ tissue culture incubator at 37°C and fed twice a week with the Neurobasal growth medium. Hippocampal neurons were transfected at days *in vitro* (DIV) 13–14 with Lipofectamine 2000 (Invitrogen) and processed at DIV 15–16.

### 4.9. Immunostaining

Coverslips containing transfected hippocampal neurons were washed in PBS and fixed with a 4% parafix solution (4% PFA and 10% sucrose in 1× PBS) for 10 min at room temperature. After washing, they were permeabilized in 0.25% Triton-X (in PBS) for 10 min, washed with PBS and blocked for 1 hour in 10% normal goat serum (NGS in PBS). The neurons were then incubated with primary antibodies in 3% NGS solution at 4°C overnight, followed by secondary antibodies in 3% NGS solution for 1 hour at room temperature. After washing, the coverslips were mounted onto glass slides with an anti-fade solution. Images were collected with a 63× oil immersion objective on a Zeiss LSM510 confocal microscope.

### 4.10. Statistics

The sample size in the figure legends represents individual puncta or neurons generated from 4 electroporated CD1 mice. Statistical analysis was performed in GraphPad Prism 10.0 using a two-tailed unpaired *t*‐test or the non-parametric Mann–Whitney test. For all statistical tests, *p *< 0.05 was considered significant. Unless stated otherwise, all data are reported as mean ± standard error of the mean (s.e.m.).

## Data Availability

The data that support the findings of this study are available from the corresponding author upon reasonable request.
